# Pediatric acute respiratory distress syndrome in children with type I - spinal muscular atrophy: a 12-year case series

**DOI:** 10.1007/s00431-025-06464-3

**Published:** 2025-09-29

**Authors:** M. Piastra, G. Zito, A. M. Orr, E. Picconi, V. Ferrari, L. Pezza, L. Marzano, T. C. Morena, G. De Rosa, R. Onesimo, M. C. Fedele, A. Tempera, O. Genovese, F. Racca, A. Varone, G. Spinazzola, D. De Luca, R. De Sanctis, M. Pane, E. Mercuri, G. Conti

**Affiliations:** 1 Emergency and Intensive Care Department, Pediatric ICU and Trauma Center, Rome, Italy; 2 Institute of Anesthesia and Intensive Care, Catholic University Medical School, Rome, Italy; 3 Intensive Care Unit, AORN “Santobono” Children Hospital, Naples, Italy; 4 Department of Pediatrics, Fondazione Policlinico Universitario “A. Gemelli” IRCCS, Rome, Italy; 5 Department of Anaesthesia and Intensive Care, Sant’Antonio E Biagio E Cesare Arrigo Hospital, Alessandria, Italy; 6 Mother and Child Department, Neonatal ICU, San Camillo-Forlanini Hospital, Rome, Italy; 7 NEMO Center for Neuromuscular Diseases, Serena Foundation and Fondazione Policlinico Universitario “A. Gemelli” IRCCS, Rome, Italy; 8 Institute of Neurology, Catholic University Medical School, Rome, Italy

**Keywords:** Pediatric ARDS, Type 1 spinal muscle atrophy, Mechanical ventilation, Complications

## Abstract

Spinal muscular atrophy type 1 (SMA-1), a disease affecting the lower motor neurons in the anterior horn cells, causes substantial respiratory morbidity and mortality in children. While recent advances in disease modifying treatments (DMTs) have improved survival and quality of life in these patients, the management and outcomes of pediatric acute respiratory distress syndrome (pARDS) in SMA-1 children have not yet been described. To report the clinical outcome and characteristics of a group of children affected by SMA-1 suffering from the most serious acute respiratory disease, i.e., pARDS, we conducted a retrospective case series of 18 SMA-1 patients with pARDS admitted to a pediatric intensive care unit over a 12-year period (2010–2021). Parameters collected included demographics, clinical and radiological data, pathology results, and respiratory support. SMA-1 patients received standard intensive care pARDS protocols, along with additional respiratory techniques such as surfactant lavage and fiberoptic bronchoscopy if necessary. Eighteen children with SMA-1, aged 2 to 42 months at the time of the ARDS episode, were included. Data collection consisted of patient demographics, clinical and radiological data, pathology results, and information about the respiratory support. The main reason for pediatric intensive care unit (PICU) admission was acute respiratory failure, mainly complicating bronchiolitis/pneumonia or gastric aspiration. The median PaO2/FiO2 ratio for all patients was 95 (IQR 85; 113), with non-survivors showing a significantly lower ratio of 67 (*p* = 0.0283). Fifteen out of 18 patients (83.3%) survived to PICU and hospital discharge. Four patients required tracheostomy and long-term mechanical ventilation, while six were discharged on NIV. All patients who survived to hospital discharge were still alive at 24-month follow-up. Mild/moderate to severe pARDS remains a clinical challenge for SMA-1 children: the present series suggests survival is achievable and current intensive pARDS protocols may be applied in the SMA-1 population.

*Conclusion*: The availability of disease modifying treatments leading to improved survival and quality of life for this severe neuromuscular disease may warrant this movement toward a more aggressive approach. The promising short-term survival and respiratory outcomes observed in this study provide valuable preliminary evidence that challenges past assumptions of futility.

**Table:** 

**What is Known:** • *Revolutionary therapies have drastically altered the natural history of type 1 spinal muscular atrophy (SMA-1), which is no longer an incurable and hopeless disease*. • *In SMA-1 infants, it has been considered futile to apply standard protocols to treat the most severe respiratory disease, namely acute respiratory distress syndrome (ARDS)*.	
**What is New:** • *This is the first case series describing pediatric acute respiratory distress syndrome (pARDS) in SMA-1 children, suggesting that standard pARDS treatment could be applied with achievable survival*.

**Supplementary Information:**

The online version contains supplementary material available at 10.1007/s00431-025-06464-3.

## Introduction

Spinal muscular atrophy (SMA) is an autosomal recessive neuromuscular disorder characterized by progressive muscle atrophy and paralysis resulting from motor neuron degeneration in the spinal cord. SMA is the second most common genetic condition and the leading genetic cause of infant mortality, with an estimated incidence of 1 in 6000–10,000 births and a carrier frequency of 1 in 35–50 [1, 2]. SMA is caused by homozygous loss of function of the survival motor neuron 1 (SMN1) gene. Based on the age of onset and severity of clinical features, SMA can be grouped into several types (type 0 to IV). The incidence of SMA I is the highest (60%), with type II and III ranging around 39%. Type 0 and IV are very rare [3, 4].

Recently, many advances have been made in the development of new therapies for neuromuscular disorders (NMDs), particularly SMA [5]. A historical breakthrough in SMA treatment came with the advent of various therapeutic approaches that increase full-length SMN protein levels. The first to receive FDA and EMA approval was Nusinersen (Spinraza™, Biogen), an antisense oligonucleotide. Another to be approved was Risdiplam (Evrysdi, Roche), a SMN2 splicing modifier. A third is Zolgensma™ (AveXis/Novartis), an adeno-associated-virus-mediated SMN1 gene replacement therapy. These treatments—available with some restrictions in different countries—have changed the natural history of SMA. Due to recent pharmacological advances [6, 7], the neurological and respiratory functions of these patients are improving compared to past decades [8]; therefore, despite their higher risk compared to the general population, management of acute respiratory failure/acute respiratory distress syndrome (ARDS) needs to be reconsidered.

Novel specific SMA therapies leading to improved survival chances and quality of life could overcome the futility that previously hindered aggressive treatments when these patients faced severe acute respiratory failure [9, 52]. Historically, chronic respiratory and neuromuscular patients have been excluded from ARDS trials being considered unlikely to survive aggressive treatments, despite parental and health workers expectations and requests [10–14]. Indeed, acute respiratory illnesses can exacerbate respiratory muscle weakness, impaired cough, and poor airway clearance, since increased secretions can produce further respiratory muscle load and acute deterioration. Severe hypoxemic respiratory failure from inhalation or superinfection, leading to pediatric ARDS (pARDS), has been described in a child with SMA-2 [15]. Yet there have been no reports of treatment protocols nor outlines of rescue strategies for these infants [16, 17]. pARDS represents the most severe form of hypoxemic respiratory failure, occurring both in previously healthy infants and children and as an acute-on-chronic complication [18]. In this study, 18 children admitted to a pediatric intensive care unit (PICU) with SMA-1 and developing pARDS, over the past decade, are presented. This retrospective case series aims to describe clinical characteristics, respiratory status at PICU discharge, and survival outcome at 24 months of SMA-1 patients with the most severe type of acute respiratory failure, i.e., pARDS.

## Methods

Patients admitted to pediatric ICU with SMA-1 and a diagnosis of pARDS in a 12-year period (2010–2021) were considered eligible for this retrospective case series. Demographic, clinical and radiological data, pathology results, and information about the respiratory support were extracted from patient’s health records. Patients were included based on the following criteria: (a) a diagnosis of SMA-1 confirmed by molecular testing [3] and (b) a diagnosis of ARDS, established according to commonly shared definitions (American-European Consensus Conference [19], Second Pediatric Acute Lung Injury Consensus Conference (PALICC-2) [20], and the Berlin definition [18]). While SMA-1 patients have chronic respiratory vulnerability due to their complex and multifactorial pathophysiology—including chronic neuromuscular weakness, restrictive lung disease, and a high risk of aspiration—only those meeting the pARDS criteria from the Second Pediatric Acute Lung Injury Consensus Conference (PALICC-2) were included in the study. These criteria include acute onset, hypoxemia, and imaging findings. A possible respiratory support prior to PICU admission, either by noninvasive ventilation (NIV) or by continuous positive airway pressure (CPAP), was reported, as well as the use of invasive ventilation during the intensive period. Length of invasive mechanical ventilation, lowest PaO2/FiO2 ratio, and use of non-conventional mechanical ventilation including high-frequency oscillatory ventilation (HFOV) and high-frequency percussive ventilation were also described. Additional treatments included bronchial lavage with surfactant, use of unilateral bronchial intubation, and bronchial blocker introduction in case of acute unilateral overinflation, need of inhaled nitric oxide, and fiberoptic bronchoscopy. Eventually, data about the patient’s condition at PICU discharge (presence of tracheostomy, need of long-term mechanical ventilation, etc.) were collected. Information is also supplied regarding the novel SMA treatments in this patient group (drug, age at introduction). CHOP INTEND (Children’s Hospital of Philadelphia Infant Test of Neuromuscular Disorders) scores, ranging from 0 to 64 (with 0 being the lowest), were reported to assess motor function prior to ARDS [51]. This provides a more comprehensive neuromuscular profile of the patients beyond the SMA-1 diagnosis alone. Similarly, we have reported nutritional status, as it may influence respiratory muscle function, immune response, and overall resilience to critical illness, offering a more comprehensive background on the patient’s clinical condition.

Descriptive statistics (median, interquartile range, mean, and standard deviation) were calculated, and comparisons between groups were made using the Student *T*-test and Pearson correlation. All statistical analysis was performed using GraphPad Prism 10.0 software.

### Ethics

This study was conducted according to the Helsinki Declaration. The institutional Ethical Committee (*Comitato Etico del Policlinico Universitario Fondazione Agostino Gemelli IRCCS*) approved the observational retrospective review of the medical files of patients with severe lower respiratory tract infections, the retrospective data collection for SMA natural history (ID 3937; May 20, 2021), and the waiver for the need for informed consent (ID 4788; April 21, 2022).

## Results

The clinical chart review identified 18 patients with SMA-1, admitted to PICU for respiratory failure, who fulfilled the diagnostic criteria for pARDS (Table 1).

**Table 1:** Clinical characteristics and outcomes of patients

Case number	1	2	3	4	5	6	7	8	9	10	11	12	13	14	15	16	17	18
Gender/age (months)	M/2.4	**M/3**	M/5.2	M/7	M/7.2	M/7.5	F/8	M/9	F/9	M/10	M/10	F/14	M/16	M/24	F/26	F/27	F/42	F36
Body weight (kg)	3.2	4	4.8	6.5	7.5	8	7.8	6.2	7.3	7.1	9	5.5	8.4	12.4	14.5	16.2	14	13
Previous respiratory support	-	NIV/HFNC	-	Noct NIV	-	-	-	Noct NIV	-	-	-	Noct NIV	Noct NIV	nasCPAP	noctNIV	noctNIV	Noct NIV	Noct NIV
Previous ICU admission	-	-	-	-	-	+	-	+	-	-	-	+	-	+	-	-	+	+
Nutritional status	SM	MM	SM	MM	AA	AA	AA	-SM	AA	MM	AA	SM-dystrophy	MM	AA	AA	AA	AA	AA
BW Z-score	−3.5	−2.9	−3.6	−2.2	−1.1	−0.7	−0.3	−3.7	−1.4	−2.8	−0.6	−6.2	−2.8	−0.2	1.4	2	−0.5	−0.6
ARF cause	Bronchiolitis	LRTI	Pneumonia	LRTI	LRTI	Bronchiol Pneum	Aspiration Pneum	Aspiration Pneum	LRTI	LRTI	bronchiolitis	LRTI	LRTI	LRTI	Aspiration Pneum	LRTI	Aspiration Pneum	LTRI
Invasive ventilation (days)	42	8	15	NA	40	22	20	16	59	7	14	12	9	7	18	11	-NA	8
NIV before iMV (hours)	72	36	24	NIV only	-	20	15	-	-	12	24	NN	6	-	24	28	NIV only	24
Additional treatments	FOB/Surf lavage BrBlock	Surf lavage	Surf lavage	na	NAVA-NIV	Surf lavage iNO	Surf lavage NAVA-NIV	no	FOB	Surf lavage	Chest drainage	Surf lavage, iNO HFPV	FOB/Surf lavage-NAVA NIV	FOB-lavage	na	NAVA-NIV	Percussion PT	Surf lavage
P/F min	85	95	115	95	170	62	92	150	67	85	110	82	90	132	95	100	110	95
O.I. max	23.1	15.4	12.8	16.8	14.5	29.5	23.5	14.6	27	23	16.1	32.4	25.6	12.8	22	19.5	15.8	22.8
pARDS subtype**	Severe	Mild/moderate	Mild/moderate	Severe	Mild/moderate	Severe	Severe	Mild/moderate	Severe	Severe	Mild/moderate	Severe	Severe	Mild/moderate	Severe	Severe	Mild/moderate	severe
Complications	RL overinfl*	RL overinfl	-	-	-	-	-	no	Massive atelect	R atelect	R empyema	Severe barotrauma, heart failure	RL overinfl	no	Difficult airway		Atelectasis (RL)	PNM
Microbiology	RSV	…	Legionella HSV	MRSa	Stenotr. Maltophil	RSV; PsAer	Str.pseumo	Enterobact	Klebsiella Pneum	-	RSV	Acromact Alcalygenes	RSV	Pseudom aeruginosa	Pseudom aeruginosa	Infl A H1N1 Str pneumo	-	Rhino, Bocavirus PseudAER
PICU stay (days)	45	32	25	17	40	23	21	30	59	21	16	5	11	17	19	16	10	10
Clinical outcome	Surv TS LTMV	TS/SB	Surv Noct NIV	Surv	Surv TS LTMV	**Dead**	Surv NIV	Surv Noct NIV	**Dead**	Surv NoctNIV	**Surv** **NIV**	**Dead**	Surv	Surv Noct NIV	Surv TS LTMV	Surv/NIV	Surv	Surv

*NIV* noninvasive ventilation, *noctNIV* overnight noninvasive ventilation, *LRTI* low-respiratory tract infection, *nasCPAP* nasal continuous positive airway pressure, *HFNC* high-flow nasal cannula, *ARF* acute respiratory failure, *IMV* invasive mechanical ventilation, *P/F* PaO2-to-FiO2 ratio, *FOB* fiberoptic bronchoscopy, *BrBlock* bronchial blocker, *iNO* inhaled nitric oxide, *HFPV* high-frequency percussive ventilation, *NAVA* neutrally adjusted ventilatory assist, *O.I.* oxygenation index, *RL overinfl* right lung overinflation, *PNM* pneumomediastinum, *MRSA* methicillin-resistant *Staphylococcus aureus*, *PsAER* *Pseudomonas aeruginosa*, *RSV* respiratory syncytial virus, *TS* tracheostomy, *LTMV* long-term mechanical ventilation, *AA* age-appropriate, *MM* moderately malnourished, *SM* severely malnourished, *M* male, *F* female, *Pneum* pneumonia, *RL* right lung

Table 2 reports the baseline conditions of these SMA-1 patients, including SMN2 gene copies, SMA-specific treatments, as well as motor function at different time points expressed as CHOP INTEND. As illustrated, the median age at diagnosis was 3.53 months, with median CHOP INTEND scores of 29.13 at diagnosis and 32.87 before the ARDS episode. The median gap between SMA diagnosis and the ARDS episode was 14.15 months.

**Table 2:** Baseline conditions of SMA-1 patients

Pt n°	SMN 2 gene copies	Age at SMA-1 diagnosis	CHOP INTEND at SMA-1 diagnosis	Last CHOP INTEND score before ARDS	Gap between diagnosis and ARDS episode	Age at SMA treatment initiation	SMA-1 specific treatment Y/N (type)
1	**2**	**3 m**	**23**	**23**	**ARDS presenting event**	**4 yrs**	**Y (nus)**
2	**2**	**4 m**	**37**	**37**	**ARDS presenting event**	**5 m**	**Y (nus)**
3	**3**	**5 m**	**40**	**36**	**3 m**	**2.1 yrs**	**Y (nus)**
4	**3**	**3 m**	**17**	**17**	**4 m**	**4 m**	**Y (nus)**
5	**3**	**3 m**	**25**	**25**	**4 m**	**5 m**	**Y (nus)**
6	**-**	**-**	**-**	**-**	**-**	**N**	**N**
7	**2**	**3 m**	**32**	**37**	**5 m**	**1.3 y**	**Y (nus)**
8	**2**	**2 m**	**-**	**-**	**7**	**6 m**	**Y (nus)**
9	**2**	**2 m**	**24**	**28**	**7 m**	**3 m**	**Y (nus)**
10	**2**	**3.5 m**	**31**	**39**	**7 m**	**5 m**	**Y (nus)**
11	**2**	**3.5 m**	**30**	**27**	**7 m**	**5 m**	**Y (zol)**
12	**-**	**5 m**	**35**	**30**	**9 m**	**7 m**	**Y (nus)**
13	**2**	**4 m**	**27**	**45**	**12 m**	**6 m**	**Y (nus)**
14	**2**	**5 m**	**32**	**40**	**19 m**	**6.5 m**	**Y (nus)**
15	**2**	**4 m**	**N**	**19**	**22 m**	**NA**	**N**
16	**2**	**3 m**	**23**	**45**	**24 m**	**2 m**	**Y (nus)**
17	**2**	**3 m**	**25**	**30**	**39 m**	**6 m**	**Y (ris)**
18	**2**	**4 m**	**36**	**48**	**32 m**	**6 m**	**Y (nus)**

*SMN* survival motor neuron, *CHOP INTEND* Children’s Hospital of Philadelphia Infant Test of Neuromuscular Disorders, *SMA* spinal muscular atrophy, *ARDS* acute respiratory distress syndrome, *NUS* Nusinersen, *RIS* Risdiplam, *ZOL* Zolgensma™, *Y* yes, *N* no, *NA* data not available

Nutritional status is better defined in Table 1, identifying age-appropriate (AA), moderately malnourished (MM), or severely malnourished (SM), according to CDC definitions (having a Z score of less than −3 for severe malnutrition).

The median age at PICU admission was 9 months (IQR 7.1; 25.0 months), median body weight 8 kg (IQR 6.6; 14.3 kg), and M/F ratio 2.25. Sixteen patients received a median of 15 days of invasive ventilation (IQR 9; 22), while two patients were given NIV only. The minimum median value for PaO_2_/FiO_2_ ratio was 95 (IQR 85; 113) for all patients and 95 (91.5; 119) for surviving patients, whereas non-survivors had 67 (62.82), *p* 0.0283. Median PICU length of stay was 21 days (IQR 14; 31) for all patients and 20 (15; 30.5) days for survivors only.

Fifteen patients (83.3%) survived to PICU and hospital discharge. Statistical differences between survivors and non-survivors were found for P/F min ratio (*p* value 0.0283), but not for age, body weight, ventilation, and PICU length, respectively.

Regarding pre-PICU admission characteristics, 10/18 patients (55.6%) had received noninvasive respiratory support, either by bilevel ventilation (nine patients) or by nasal CPAP (one patient). No patient had a percutaneous endoscopic gastrostomy at the time of ARDS treatment in PICU, whereas nutritional status assessment data are inserted in Table 1.

**Fig. 1:**
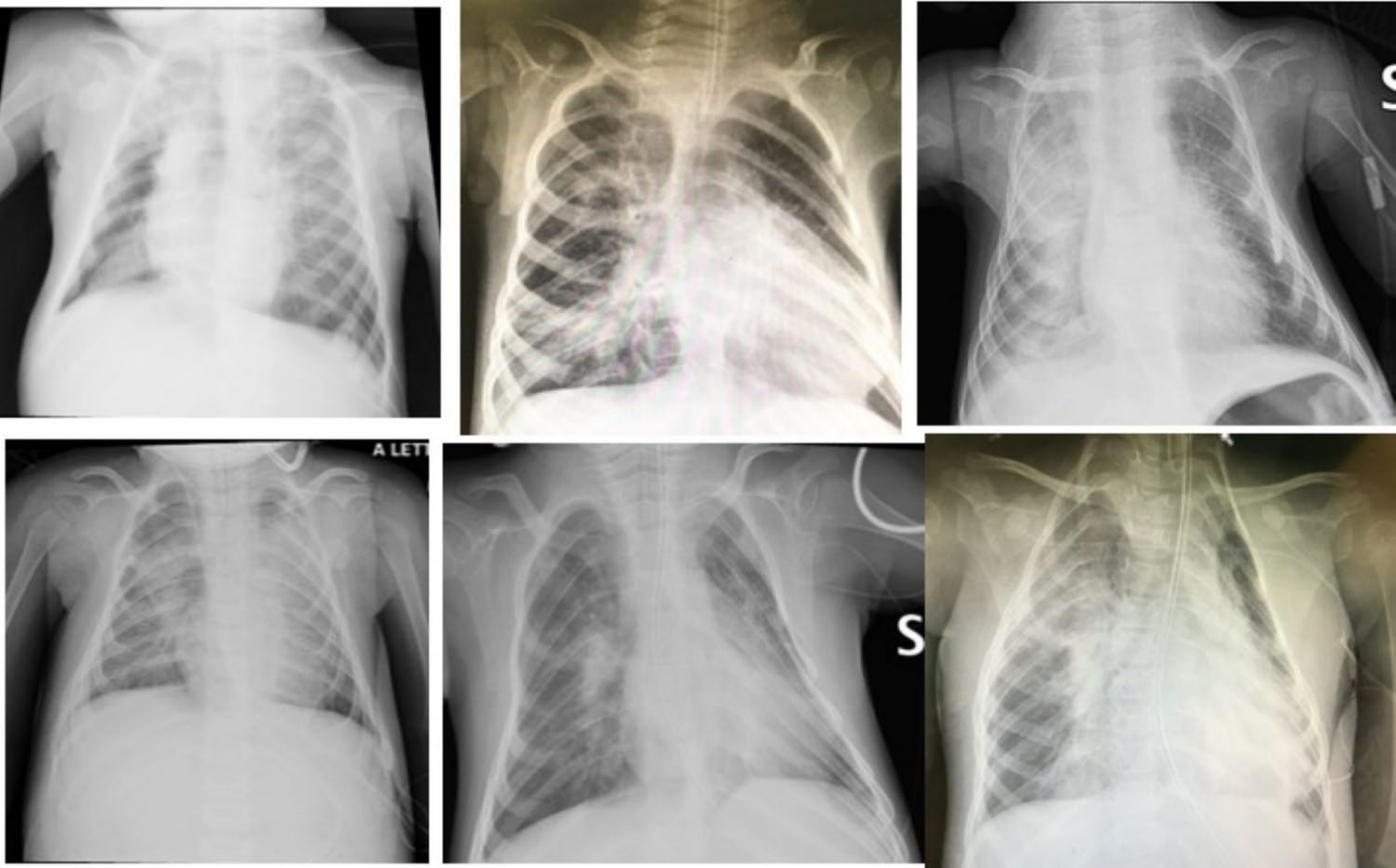
Chest X-rays appearance of type 1 SMA patients affected by ARDS

**Fig. 2:**
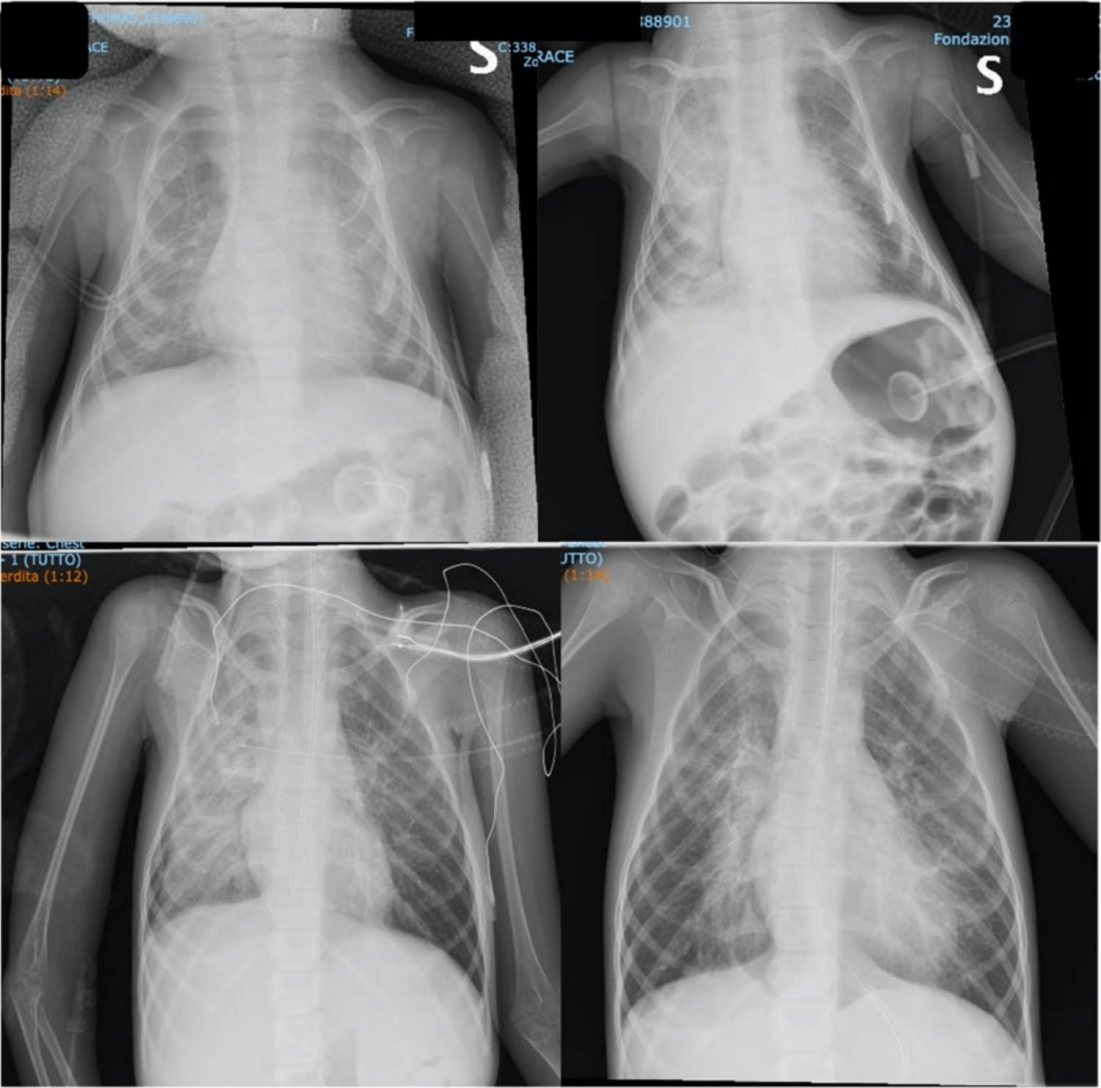
Chest radiological features of SMA-1 patients managed exclusively with NIV

All but two patients underwent invasive mechanical ventilation (Fig. 1); chest imaging of noninvasively treated patients is shown in Fig. 2. A pulmonary infection triggered ARDS in most patients, while four patients showed clear signs of aspiration pneumonia. Beyond conventional ventilatory support, several additional treatments were required (surfactant lavage (10 pts), iNO (2 pts), NAVA-NIV (4 pts), fiberoptic bronchoscopy (4 pts), and high-frequency percussive ventilation (1 pt).

Several viral or bacterial respiratory pathogens were isolated from nasal/pharyngeal swabs, sputum, or broncho-alveolar lavage; in three patients, a coinfection of a viral and bacterial agent was found (Table 1).

PICU length of stay was found to be inversely correlated to age and weight and directly correlated to ventilation requirement, expressed as the length of invasive mechanical ventilation (Graph 1).

Three patients died during PICU stay: two from refractory hypoxemia (including one with massive atelectasis), and one following severe barotrauma, heart failure, and persistent hypoxemia after emergency transfer in critical condition (Table 1).

Four out of 15 surviving patients received a surgical tracheostomy, and long-term mechanical ventilation was started, while six patients were discharged from PICU under continuous NIV. All surviving children were alive at 24-month follow-up and continued their previously initiated therapeutic regimen, including long-term intrathecal administration of Nusinersen where applicable.

## Discussion

Patients with SMA-1 are susceptible to respiratory failure development, due to the early respiratory mechanics deterioration and chest deformity, thereby inducing airway secretion retention and recurrent atelectasis. The severe infantile form of SMA-1 is characterized by clinical manifestation of the disease before the age of 6 months. Its onset is acute and rapidly progressive [1, 4, 21, 52].

Besides viral respiratory infections, both gastric aspiration and bacterial superinfections may eventually precipitate the clinical picture of pARDS.

To date, the pARDS management approach involves the use of “protective ventilation” aimed at keeping the plateau pressure (as detected by the ventilator software when an inspiratory pause is applied) under 28–30 cmH2O and the driving pressure (difference between plateau pressure and PEEP) under 14 cmH2O. Therefore, when the pulmonary compliance is markedly reduced in the inflamed lung, tidal volumes as low as 5–6 mL/kg should be required. Larger tidal volumes can be applied whenever pulmonary compliance is less reduced, mostly in the recovery phase [20].

pARDS in SMA-1 patients is a rare and difficult situation to treat, especially as their respiratory failure is complex and multifactorial. The lack of standardized guidelines complicates decision-making, leaving treatment approaches largely dependent on institutional expertise. As a general rule, there are no treatment strategies for the acute hypoxemic phase of pARDS specifically designed for neuromuscular patients, whereas their weaning process may be prolonged and complicated by multiple extubation failure episodes. In fact, meticulous attention to ventilator management, airway clearance, nutrition/fluid management, and sedation is fundamental for optimal treatment of respiratory failure in this population. Extubation protocols for neuromuscular patients have been available for at least two decades, though not specifically designed for patients recovering from an ARDS episode [22]. The integrated use of multiple airway clearance tools should be advocated (recruitment of peripheral secretions using high-frequency devices, i.e., percussive and high-frequency chest wall oscillation, combined with central airway clearance with mechanical in-exsufflation): this approach is currently used bedside in our PICU.

This study also reports a multi-step evaluation of muscular performance of this SMA-1 patient subset, of particular importance in the recovery phase of ARDS (while controlled ventilation is warranted in the acute one, aiming at lung recruitment and oxygenation improvement): this data is of ultimate importance, as it can allow a strict cooperation between respiratory therapists and intensivists.

Some preventive measures can be of fundamental importance in the respiratory care of SMA-1 patients: first, the preemptive introduction of NIV support (as bilevel ventilation, frequently overnight) for preventing progressive chest bell-shape deformity [23], long before respiratory failure occurs, second, the extensive use of gastrostomy, achieving a better nutritional and immunological status and reducing the aspiration episodes when bulbar involvement is present [8]. Preemptive gastrostomy placement in our patients could be delayed due to parental reluctance to discontinue oral feeding, despite the well-established advantages of gastrostomy in patients with neuromuscular diseases such as SMA-1. In fact, among patients who developed ARDS due to aspiration pneumonia, none had a prior gastrostomy.

In the past, there has been considerable debate regarding the ethics of invasive mechanical ventilation for SMA-1 complicated by severe respiratory failure, since they could become ‘‘locked-in’’ as the disease progressed, leaving them unable to communicate pain or discomfort [24, 25]. Moreover, intensivists are more likely to see children when they are suffering from an acute respiratory illness and may not appreciate their quality of life in relative health. Recently, the availability of promising therapies for motor neuron diseases [26, 27] could have changed the attitude of the intensivist toward SMA-1 patients dealing with acute severe respiratory conditions. Moreover, real-world experience suggests that SMA-specific drugs can help stabilize motor and respiratory functions or even alter the disease phenotype, unlike the progression in the clinical course seen in the past [28, 29].

A noninvasive respiratory management approach can be considered a first-line treatment for acute respiratory failure in patients with neuromuscular diseases; as reported by Chen et al., NIV combined with mechanical in-exsufflation cough assist has the potential to reduce the need for intubation and to shorten PICU length of stay [30, 31]. Moreover, NIV can contribute to prevent re-intubation in SMA children weaned from invasive mechanical ventilation [32]. However, children suffering from SMA-1 accounted for a small minority in such retrospective studies; acute respiratory failure is usually induced by pneumonia or atelectasis; alveolar hypoventilation—resulting in exacerbation of hypercapnia—is usually predominant, while clinical reports of ARDS in SMA patients are rare, due both to the paucity and the futility issues in the past. Our case series can represent a basis for ARDS treatment in such neuromuscular patients having severe lung function compromise.

pARDS is characterized by acute development of clinically significant hypoxemia, concurrently with the appearance of bilateral diffuse pulmonary infiltrates. The approach to severe hypoxemic respiratory failure in SMA-1 patients was similar to that for previously healthy children. The current standard management to mechanical ventilation in every child with pARDS includes bedside respiratory mechanics assessment, low tidal volumes with permissive hypercapnia, adequate PEEP to optimize lung recruitment, and maintaining plateau pressures to less than 28 cmH_2_0 [33, 34]. Inhaled nitric oxide, surfactant lavage, and nonconventional ventilation techniques may be considered in severe ARDS, not responding to first-line therapeutic strategies [35].

Protective ventilation is essential to avoid ventilator-associated lung injury; nonetheless, SMA patients are at increased risk for atelectasis secondary to suboptimal airway clearance and mucus plugging [17]. In our experience, endotracheal instillation of porcine surfactant was used in nine patients, according to a shared protocol [36]; although the role of this drug in pARDS is controversial, it may improve lung compliance, foster pulmonary recruitment, and, through its cleaning effect, remove inflammatory mediators and cell debris [37, 38]. Moreover, both 2015 Cochrane review and several authors support the use of natural surfactant in bronchiolitis-associated ARDS [38, 39]. A possible explanation for the failure of surfactant instillation in ARDS in older children and adults has been suggested by studies regarding the airway distribution of this agent [40].

Use of flexible fiberoptic bronchoscopy and innovative approaches represents a useful support in difficult secretions management, since it allows removal of endobronchial secretions and atelectasis resolution: to date, its application in SMA-1 patients is rarely described [41, 42]. When one-side lung collapse resulted intractable leading to marked contralateral overinflation during positive pressure ventilation, unilateral bronchial intubation or, eventually, selective placement of a bronchial blocker into the main bronchus of the enlarged lung has been adopted to achieve pulmonary re-expansion.

Lower respiratory tract infections represent the leading cause of unplanned PICU admission for SMA-1 patients, as well as the most common trigger of pARDS. While a viral infection is often the root cause of ARF in SMA-1 patients, continuous retention of airway secretions increases the risk of chronic pathogenic bacterial colonization, especially from *Staphylococcus aureus*, *Pseudomonas aeruginosa*, or other Gram-negative species, which may therefore evolve into pneumonia [43]; interestingly, one patient in our series was infected with influenza swine-A H1N1, a virus typically associated with severe hypoxemia and rapid progression toward ARDS [44], and *Legionella pneumophila* was also observed.

Reported mortality rate of pediatric ARDS from recent studies is about 24–27% [45, 46]: however, while patients with neuromuscular diseases represent, sometimes, an appreciable part of pARDS population (17% in PARDIE study [46]), SMA-1 children’s expectancy to survive pARDS is difficult to estimate; actually, in the current literature, descriptions of the clinical management of pARDS in SMA children are often limited to single case reports [15]. In the recovery phase, an integrated respiratory management program, including NIV application, use of mechanical insufflation-exsufflation, and chest physical therapy, may allow to avoid tracheostomy for most children [32]. Neurally adjusted ventilatory assist (NAVA) may represent a valuable tool for weaning, virtually superior to noninvasive flow-triggered pressure support, since the optimal neuroventilatory coupling allows to markedly improve patient-ventilator interaction [47–49]: this feature can be fundamental in the weaning phase of severe ARDS [49, 50].

A limitation of our study is its retrospective design which limits the ability to establish causal relationships or control confounding variables. The management of patients in our study is based on real-life clinical experience rather than standardized protocols. As a two-center study, the findings may not be generalizable to other institutions, particularly due to the absence of data in the literature and guidelines for pARDS in SMA-1 patients. Additionally, the sample size is small, reflecting the rarity of both SMA-1 and severe pARDS in this population, which limits the statistical power of our observations.

Another important limitation is the focus on short-term outcomes. While we report survival and initial respiratory outcomes following PICU admission, long-term follow-up data on functional respiratory status, quality of life, and neuromuscular progression are limited. This makes it difficult to fully assess the impact of an intensive approach beyond the acute phase.

The focus on short-term outcomes also raises ethical concerns, as the long-term consequences of aggressive interventions remain uncertain. Given the complexity of these decisions, healthcare teams should carefully discuss options with families, ensuring that treatments are appropriate based on individual situations. This issue reflects an ongoing and complex debate in the management of SMA patients, where the overall prognosis for the disease has improved but long-term outcomes are still uncertain.

Future studies with larger, multicenter cohorts and longitudinal follow-up are necessary to evaluate the long-term benefits and challenges of aggressive respiratory management in SMA-1 patients with pARDS.

## Conclusion

This retrospective case series reports the clinical outcomes and characteristics of children with SMA-1 who developed pARDS. Our findings describe how the implemented therapeutic approach and rescue strategies led to respiratory improvement and survival.

pARDS in SMA-1 children represents a clinical challenge for the pediatric intensivist. Our findings suggest that survival is achievable and that aggressive treatment may be warranted, in light of therapeutic advancements that have improved the overall prognosis of SMA-1. The promising short-term survival and respiratory outcomes observed in this study provide valuable preliminary evidence that challenges past assumptions of futility.

This observation prompts two key considerations. First, it underscores the need for further studies to validate and support a shift toward a more aggressive approach in managing SMA-1 patients with pARDS. Second, it encourages reflection on current medical practice, highlighting a growing tendency toward more invasive interventions for SMA1 patients.

## Supplementary Information

Below is the link to the electronic supplementary material.

ESM 1 Supplementary Material 1 (DOCX 39.1 KB)

## Data Availability

The data that support the findings of this study are available on request from the corresponding author. The data are not publicly available due to privacy or ethical restrictions.
