# Supplementary material for: Pediatric acute respiratory distress syndrome in children with type I - spinal muscular atrophy: a 12-year case series
Source: Eur J Pediatr. 2025 Sep 29;184(10):649. doi: 10.1007/s00431-025-06464-3 (PMC12477088; doi:10.1007/s00431-025-06464-3)
Supplement: Supplementary file 1 — Supplementary Material 1 (DOCX 39.1 KB) [file 431_2025_6464_MOESM1_ESM.docx]

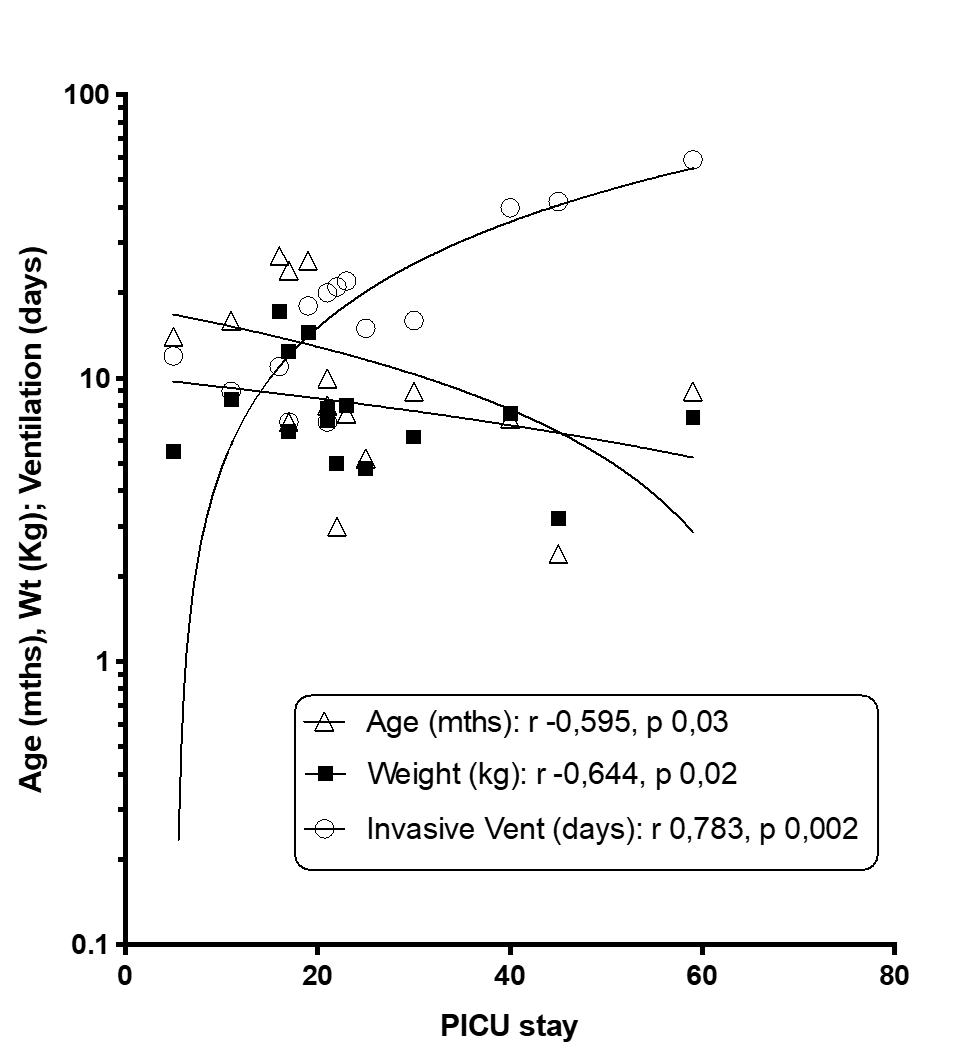


**Graph 1**. Correlation Between PICU length of stay, age/body weight and ventilation requirement in SMA-1 patients with pARDS
